# 2-(2-{[2-(2-Pyridylcarbon­yl)hydrazono]meth­yl}phen­oxy)acetic acid

**DOI:** 10.1107/S160053680905082X

**Published:** 2009-12-04

**Authors:** Bao-Yu Liu, Zheng Liu, Guo-Rui Wang

**Affiliations:** a College of Chemical and Biological Engineering (Guilin University of Technology), Guilin 541004, People’s Republic of China

## Abstract

In the title compound, C_15_H_13_N_3_O_4_, the pyridine and benzene rings are nearly coplanar [dihedral angle = 4.92 (12)°]. The maximum deviation from the best least-squares plane calculated for the main mol­ecular skeleton is 0.1722 (1) Å for the carbonyl O atom. In the crystal, inter­molecular O—H⋯O hydrogen bonds connect the mol­ecules into a chain, while π–π stacking inter­actions between the pyridine and benzene rings [centroid–centroid distance = 3.9162 (8) Å and offset angle = 27.20°] complete a two-dimensional network.

## Related literature

For Schiff bases complexes containing (*O*-oxyacetic acid)benzaldehyde, see: Wu *et al.* (2003 ▶). 
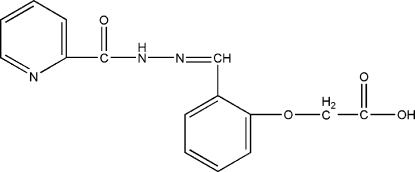


## Experimental

### 

#### Crystal data

C_15_H_13_N_3_O_4_
*M* *_r_* = 299.28
Monoclinic, 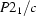

*a* = 8.871 (2) Å
*b* = 9.042 (2) Å
*c* = 17.389 (4) Å
β = 94.765 (3)°
*V* = 1390.0 (5) Å^3^
*Z* = 4
Mo *K*α radiation
μ = 0.11 mm^−1^
*T* = 296 K
0.16 × 0.15 × 0.04 mm

#### Data collection

Bruker APEXII CCD diffractometer
Absorption correction: multi-scan (*SADABS*; Bruker, 1998 ▶) *T*_min_ = 0.983, *T*_max_ = 0.996
11832 measured reflections
3194 independent reflections
1512 reflections with *I* > 2σ(*I*)
*R*_int_ = 0.074

#### Refinement

*R*[*F* ^2^ > 2σ(*F* ^2^)] = 0.050
*wR*(*F* ^2^) = 0.125
*S* = 0.99
3194 reflections
200 parameters
H-atom parameters constrained
Δρ_max_ = 0.20 e Å^−3^
Δρ_min_ = −0.20 e Å^−3^

### 

Data collection: *APEX2* (Bruker, 2004 ▶); cell refinement: *SAINT* (Bruker, 2004 ▶); data reduction: *SAINT*; program(s) used to solve structure: *SHELXS97* (Sheldrick, 2008 ▶); program(s) used to refine structure: *SHELXL97* (Sheldrick, 2008 ▶); molecular graphics: *SHELXTL* (Sheldrick, 2008 ▶); software used to prepare material for publication: *SHELXTL*.

## Supplementary Material

Crystal structure: contains datablocks global, I. DOI: 10.1107/S160053680905082X/kp2238sup1.cif

Structure factors: contains datablocks I. DOI: 10.1107/S160053680905082X/kp2238Isup2.hkl

Additional supplementary materials:  crystallographic information; 3D view; checkCIF report

## Figures and Tables

**Table 1:** Hydrogen-bond geometry (Å, °)

*D*—H⋯*A*	*D*—H	H⋯*A*	*D*⋯*A*	*D*—H⋯*A*
O4—H4*A*⋯O1^i^	0.82	1.83	2.642 (2)	171

Symmetry code: (i) 
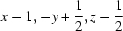
.

**Fig. 1.**
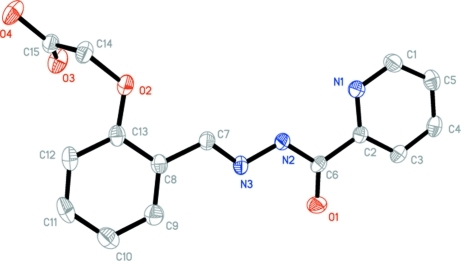
The molecular structure of (I), showing 30% probability displacement ellipsoids for non-H atoms. H atoms bound to C and N have been omitted.

**Fig. 2.**
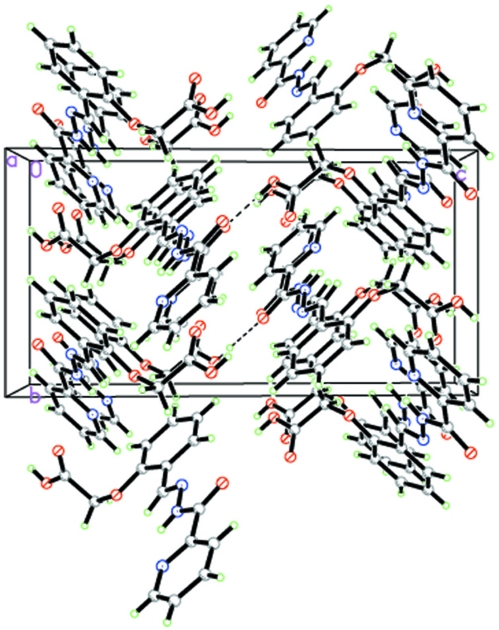
Partial packing diagram showing a hydrogen-bonded chain running along the a axis.

## supplementary crystallographic information

### Comment

The molecular structure of (I) (Fig.1) reveals the nearly planar system; the dihedral angle between the pyridine and benzene rings is 4.923°. An intermolecular O–H···O hydrogen bond connects molecules into a chain (Table 1, Fig.2). The pyridine ring (1-x,-1/2+y,1/2-z) is parallel to the benzene ring (-x,-1/2+y,1/2-z) with a perpendicular distance of 3.3239 Å: a centroid–centroid = 3.9162 (8) Å and an offset angle = 27.197° (calculated as the angle between the line through the two centroids of the pyridine ring and the benzene ring and a normal to the pyridine plane. Thus pi–pi stacking interactions complete a two dimensional network (Fig.2).

### Experimental

A methanol solution (10 ml) was added to an acetone solution (10 ml) of the 2-(2-methoxyacetic acid)benzaldehyde picoloylhydrazone (0.5 mmol). After stirring at 35\ % for 2 h, crystals of the title compound were obtained by slow evaporation of the mixture at room temperature.

### Refinement

H atoms were placed at calculated positions (C–H = 0.93 Å and O—H = 0.82 Å) and were included in the refinement in the riding model approximation, with *U*_iso_(H) = 1.2*U*_eq_(C, N) and [*U*_iso_(H) = 1.5*U*_eq_(O)].

### Crystal data

**Table:**

C_15_H_13_N_3_O_4_	*F*(000) = 624
*M**_r_* = 299.28	*D*_x_ = 1.430 Mg m^−^^3^
Monoclinic, *P*2_1_/*c*	Mo *K*α radiation, λ = 0.71073 Å
Hall symbol: -P 2ybc	Cell parameters from 1231 reflections
*a* = 8.871 (2) Å	θ = 2.3–20.3°
*b* = 9.042 (2) Å	µ = 0.11 mm^−^^1^
*c* = 17.389 (4) Å	*T* = 296 K
β = 94.765 (3)°	Block, colourless
*V* = 1390.0 (5) Å^3^	0.16 × 0.15 × 0.04 mm
*Z* = 4	

### Data collection

**Table:**

Bruker APEXII CCD diffractometer	3194 independent reflections
Radiation source: fine-focus sealed tube	1512 reflections with *I* > 2σ(*I*)
graphite	*R*_int_ = 0.074
φ and ω scans	θ_max_ = 27.5°, θ_min_ = 2.3°
Absorption correction: multi-scan (*SADABS*; Bruker, 1998)	*h* = −11→11
*T*_min_ = 0.983, *T*_max_ = 0.996	*k* = −11→10
11832 measured reflections	*l* = −22→22

### Refinement

**Table:**

Refinement on *F*^2^	Primary atom site location: structure-invariant direct methods
Least-squares matrix: full	Secondary atom site location: difference Fourier map
*R*[*F*^2^ > 2σ(*F*^2^)] = 0.050	Hydrogen site location: inferred from neighbouring sites
*wR*(*F*^2^) = 0.125	H-atom parameters constrained
*S* = 0.99	*w* = 1/[σ^2^(*F*_o_^2^) + (0.0467*P*)^2^ + 0.0303*P*] where *P* = (*F*_o_^2^ + 2*F*_c_^2^)/3
3194 reflections	(Δ/σ)_max_ < 0.001
200 parameters	Δρ_max_ = 0.20 e Å^−^^3^
0 restraints	Δρ_min_ = −0.19 e Å^−^^3^

### Special details

**Table:**

Geometry. All e.s.d.'s (except the e.s.d. in the dihedral angle between two l.s. planes) are estimated using the full covariance matrix. The cell e.s.d.'s are taken into account individually in the estimation of e.s.d.'s in distances, angles and torsion angles; correlations between e.s.d.'s in cell parameters are only used when they are defined by crystal symmetry. An approximate (isotropic) treatment of cell e.s.d.'s is used for estimating e.s.d.'s involving l.s. planes.
Refinement. Refinement of *F*^2^ against ALL reflections. The weighted *R*-factor *wR* and goodness of fit *S* are based on *F*^2^, conventional *R*-factors *R* are based on *F*, with *F* set to zero for negative *F*^2^. The threshold expression of *F*^2^ > σ(*F*^2^) is used only for calculating *R*-factors(gt) *etc*. and is not relevant to the choice of reflections for refinement. *R*-factors based on *F*^2^ are statistically about twice as large as those based on *F*, and *R*- factors based on ALL data will be even larger.

### Fractional atomic coordinates and isotropic or equivalent isotropic displacement parameters (Å^2^)

**Table:**

	*x*	*y*	*z*	*U*_iso_*/*U*_eq_	
N1	0.5796 (2)	0.6090 (2)	0.33901 (11)	0.0446 (5)	
C2	0.6090 (3)	0.5119 (2)	0.39612 (13)	0.0353 (6)	
C3	0.7443 (3)	0.5054 (3)	0.44083 (14)	0.0436 (6)	
H3	0.7596	0.4364	0.4803	0.052*	
C4	0.8563 (3)	0.6037 (3)	0.42557 (15)	0.0513 (7)	
H4	0.9488	0.6027	0.4549	0.062*	
C5	0.8296 (3)	0.7032 (3)	0.36644 (16)	0.0538 (7)	
H5	0.9044	0.7692	0.3543	0.065*	
C6	0.4850 (2)	0.4041 (3)	0.40971 (13)	0.0371 (6)	
C7	0.1114 (3)	0.3732 (2)	0.33134 (13)	0.0409 (6)	
H7	0.1103	0.4555	0.2993	0.049*	
C8	−0.0249 (3)	0.2837 (3)	0.33563 (14)	0.0400 (6)	
C9	−0.0354 (3)	0.1807 (3)	0.39414 (15)	0.0514 (7)	
H9	0.0465	0.1669	0.4304	0.062*	
C10	−0.1651 (3)	0.0984 (3)	0.39943 (16)	0.0598 (8)	
H10	−0.1709	0.0302	0.4391	0.072*	
C11	−0.2859 (3)	0.1183 (3)	0.34527 (17)	0.0600 (8)	
H11	−0.3733	0.0625	0.3484	0.072*	
C12	−0.2790 (3)	0.2190 (3)	0.28689 (15)	0.0497 (7)	
H12	−0.3611	0.2309	0.2506	0.060*	
C13	−0.1494 (3)	0.3035 (3)	0.28189 (14)	0.0412 (6)	
C14	−0.2598 (3)	0.4491 (3)	0.17649 (14)	0.0487 (7)	
H14A	−0.2442	0.5471	0.1559	0.058*	
H14B	−0.3484	0.4531	0.2056	0.058*	
C15	−0.2888 (3)	0.3425 (3)	0.11069 (14)	0.0426 (6)	
C1	0.6908 (3)	0.7033 (3)	0.32572 (15)	0.0541 (7)	
H1	0.6727	0.7729	0.2866	0.065*	
N2	0.3553 (2)	0.4263 (2)	0.36523 (10)	0.0414 (5)	
H2	0.3495	0.4971	0.3321	0.050*	
N3	0.2323 (2)	0.3368 (2)	0.37230 (11)	0.0401 (5)	
O1	0.50069 (18)	0.30437 (19)	0.45682 (10)	0.0563 (5)	
O2	−0.13215 (17)	0.40989 (18)	0.22698 (9)	0.0486 (5)	
O3	−0.21338 (19)	0.2346 (2)	0.10071 (10)	0.0603 (5)	
O4	−0.4074 (2)	0.38551 (19)	0.06633 (10)	0.0612 (6)	
H4A	−0.4261	0.3245	0.0320	0.092*	

### Atomic displacement parameters (Å^2^)

**Table:**

	*U*^11^	*U*^22^	*U*^33^	*U*^12^	*U*^13^	*U*^23^
N1	0.0383 (12)	0.0473 (13)	0.0471 (13)	−0.0034 (10)	−0.0024 (10)	0.0064 (11)
C2	0.0341 (13)	0.0374 (14)	0.0346 (14)	0.0014 (11)	0.0031 (11)	−0.0057 (11)
C3	0.0408 (15)	0.0490 (16)	0.0399 (14)	0.0042 (12)	−0.0036 (12)	−0.0018 (12)
C4	0.0336 (14)	0.0615 (18)	0.0570 (18)	−0.0024 (13)	−0.0074 (12)	−0.0081 (15)
C5	0.0400 (16)	0.0540 (18)	0.067 (2)	−0.0075 (13)	0.0040 (14)	−0.0046 (15)
C6	0.0332 (13)	0.0431 (15)	0.0343 (14)	0.0066 (12)	−0.0012 (10)	−0.0025 (12)
C7	0.0373 (14)	0.0406 (15)	0.0437 (15)	−0.0033 (12)	−0.0022 (11)	−0.0015 (12)
C8	0.0373 (14)	0.0376 (14)	0.0450 (15)	−0.0018 (11)	0.0024 (12)	−0.0069 (12)
C9	0.0504 (17)	0.0522 (17)	0.0516 (17)	−0.0016 (14)	0.0030 (13)	−0.0037 (14)
C10	0.0646 (19)	0.0526 (18)	0.064 (2)	−0.0104 (15)	0.0133 (16)	0.0011 (15)
C11	0.0496 (18)	0.0554 (19)	0.077 (2)	−0.0225 (14)	0.0168 (16)	−0.0168 (16)
C12	0.0350 (15)	0.0559 (17)	0.0579 (18)	−0.0085 (13)	0.0024 (13)	−0.0154 (15)
C13	0.0377 (14)	0.0415 (15)	0.0444 (15)	−0.0029 (12)	0.0034 (12)	−0.0129 (13)
C14	0.0409 (15)	0.0457 (16)	0.0571 (17)	0.0030 (12)	−0.0092 (13)	−0.0047 (13)
C15	0.0388 (14)	0.0437 (15)	0.0441 (15)	−0.0020 (13)	−0.0033 (12)	−0.0009 (13)
C1	0.0487 (17)	0.0505 (17)	0.0626 (19)	−0.0043 (14)	0.0027 (14)	0.0102 (15)
N2	0.0340 (11)	0.0422 (12)	0.0460 (12)	−0.0027 (10)	−0.0082 (9)	0.0068 (10)
N3	0.0322 (11)	0.0396 (12)	0.0473 (13)	−0.0035 (9)	−0.0027 (10)	−0.0030 (9)
O1	0.0467 (11)	0.0625 (12)	0.0580 (12)	0.0017 (9)	−0.0051 (9)	0.0223 (10)
O2	0.0398 (10)	0.0541 (11)	0.0496 (11)	−0.0060 (8)	−0.0093 (8)	−0.0013 (9)
O3	0.0521 (11)	0.0617 (13)	0.0654 (13)	0.0137 (10)	−0.0050 (9)	−0.0172 (10)
O4	0.0642 (12)	0.0577 (13)	0.0569 (13)	0.0116 (10)	−0.0242 (10)	−0.0138 (9)

### Geometric parameters (Å, °)

**Table:**

N1—C2	1.335 (3)	C9—H9	0.9300
N1—C1	1.338 (3)	C10—C11	1.378 (4)
C2—C3	1.376 (3)	C10—H10	0.9300
C2—C6	1.502 (3)	C11—C12	1.369 (3)
C3—C4	1.375 (3)	C11—H11	0.9300
C3—H3	0.9300	C12—C13	1.389 (3)
C4—C5	1.372 (3)	C12—H12	0.9300
C4—H4	0.9300	C13—O2	1.373 (3)
C5—C1	1.369 (3)	C14—O2	1.419 (2)
C5—H5	0.9300	C14—C15	1.502 (3)
C6—O1	1.219 (3)	C14—H14A	0.9700
C6—N2	1.347 (2)	C14—H14B	0.9700
C7—N3	1.280 (3)	C15—O3	1.204 (3)
C7—C8	1.462 (3)	C15—O4	1.311 (3)
C7—H7	0.9300	C1—H1	0.9300
C8—C9	1.388 (3)	N2—N3	1.372 (2)
C8—C13	1.398 (3)	N2—H2	0.8600
C9—C10	1.380 (3)	O4—H4A	0.8200
			
C2—N1—C1	116.5 (2)	C12—C11—C10	120.9 (2)
N1—C2—C3	123.7 (2)	C12—C11—H11	119.6
N1—C2—C6	116.3 (2)	C10—C11—H11	119.6
C3—C2—C6	119.9 (2)	C11—C12—C13	120.1 (2)
C4—C3—C2	118.3 (2)	C11—C12—H12	120.0
C4—C3—H3	120.9	C13—C12—H12	120.0
C2—C3—H3	120.9	O2—C13—C12	124.7 (2)
C5—C4—C3	119.1 (2)	O2—C13—C8	115.3 (2)
C5—C4—H4	120.5	C12—C13—C8	120.0 (2)
C3—C4—H4	120.5	O2—C14—C15	112.85 (19)
C1—C5—C4	118.7 (3)	O2—C14—H14A	109.0
C1—C5—H5	120.7	C15—C14—H14A	109.0
C4—C5—H5	120.7	O2—C14—H14B	109.0
O1—C6—N2	122.7 (2)	C15—C14—H14B	109.0
O1—C6—C2	122.8 (2)	H14A—C14—H14B	107.8
N2—C6—C2	114.5 (2)	O3—C15—O4	125.7 (2)
N3—C7—C8	119.2 (2)	O3—C15—C14	124.6 (2)
N3—C7—H7	120.4	O4—C15—C14	109.7 (2)
C8—C7—H7	120.4	N1—C1—C5	123.7 (3)
C9—C8—C13	118.6 (2)	N1—C1—H1	118.2
C9—C8—C7	121.0 (2)	C5—C1—H1	118.2
C13—C8—C7	120.4 (2)	C6—N2—N3	120.6 (2)
C10—C9—C8	121.2 (2)	C6—N2—H2	119.7
C10—C9—H9	119.4	N3—N2—H2	119.7
C8—C9—H9	119.4	C7—N3—N2	115.7 (2)
C9—C10—C11	119.3 (3)	C13—O2—C14	118.43 (18)
C9—C10—H10	120.4	C15—O4—H4A	109.5
C11—C10—H10	120.4		
			
C1—N1—C2—C3	0.4 (3)	C11—C12—C13—O2	−178.3 (2)
C1—N1—C2—C6	−178.7 (2)	C11—C12—C13—C8	1.2 (4)
N1—C2—C3—C4	−0.5 (4)	C9—C8—C13—O2	178.4 (2)
C6—C2—C3—C4	178.5 (2)	C7—C8—C13—O2	0.2 (3)
C2—C3—C4—C5	−0.4 (4)	C9—C8—C13—C12	−1.2 (3)
C3—C4—C5—C1	1.5 (4)	C7—C8—C13—C12	−179.4 (2)
N1—C2—C6—O1	175.4 (2)	O2—C14—C15—O3	0.0 (4)
C3—C2—C6—O1	−3.7 (3)	O2—C14—C15—O4	−179.8 (2)
N1—C2—C6—N2	−4.2 (3)	C2—N1—C1—C5	0.8 (4)
C3—C2—C6—N2	176.6 (2)	C4—C5—C1—N1	−1.7 (4)
N3—C7—C8—C9	13.7 (3)	O1—C6—N2—N3	1.0 (3)
N3—C7—C8—C13	−168.2 (2)	C2—C6—N2—N3	−179.28 (19)
C13—C8—C9—C10	0.4 (4)	C8—C7—N3—N2	−179.88 (19)
C7—C8—C9—C10	178.5 (2)	C6—N2—N3—C7	175.3 (2)
C8—C9—C10—C11	0.4 (4)	C12—C13—O2—C14	7.3 (3)
C9—C10—C11—C12	−0.4 (4)	C8—C13—O2—C14	−172.3 (2)
C10—C11—C12—C13	−0.4 (4)	C15—C14—O2—C13	−81.0 (3)

### Hydrogen-bond geometry (Å, °)

**Table:**

*D*—H···*A*	*D*—H	H···*A*	*D*···*A*	*D*—H···*A*
O4—H4A···O1^i^	0.82	1.83	2.642 (2)	171

Symmetry codes: (i) *x*−1, −*y*+1/2, *z*−1/2.
